# CpG sites associated with *NRP1, NRXN2* and miR-29b-2 are hypomethylated in monocytes during ageing

**DOI:** 10.1186/1742-4933-11-1

**Published:** 2014-01-09

**Authors:** Liina Tserel, Maia Limbach, Mario Saare, Kai Kisand, Andres Metspalu, Lili Milani, Pärt Peterson

**Affiliations:** 1 Molecular Pathology, Institute of Biomedicine and Translational Medicine, University of Tartu, 19 Ravila St, Tartu 50411, Estonia; 2 Estonian Genome Centre, University of Tartu, Tartu, Estonia

**Keywords:** Monocytes, DNA methylation, Ageing

## Abstract

**Background:**

Ageing affects many components of the immune system, including innate immune cells like monocytes. They are important in the early response to pathogens and for their role to differentiate into macrophages and dendritic cells. Recent studies have revealed significant age-related changes in genomic DNA methylation in peripheral blood mononuclear cells, however information on epigenetic changes in specific leukocyte subsets is still lacking. Here, we aimed to analyse DNA methylation in purified monocyte populations from young and elderly individuals.

**Findings:**

We analysed the methylation changes in monocytes purified from young and elderly individuals using the HumanMethylation450 BeadChip array. Interestingly, we found that among 26 differentially methylated CpG sites, the majority of sites were hypomethylated in elderly individuals. The most hypomethylated CpG sites were located in neuropilin 1 (*NRP1*; cg24892069) and neurexin 2 (*NRXN2;* cg27209729) genes, and upstream of miR-29b-2 gene (cg10501210). The age-related hypomethylation of these three sites was confirmed in a separate group of young and elderly individuals.

**Conclusions:**

We identified significant age-related hypomethylation in human purified monocytes at CpG sites within the regions of *NRP1*, *NRXN2* and miR-29b-2 genes.

## Main text

Innate and adaptive immune responses are affected by ageing. Elderly people have a decreased ability to maintain basic tissue homeostasis, impaired vaccination responses and an increased risk for infectious diseases, particularly influenza virus [1-4]. A diverse range of age-associated changes has been reported in human innate immune cells [3,5], which are important during the early response to pathogens. Monocytes, which are circulating cells that originate from myeloid precursors, are the precursors of tissue macrophages and dendritic cells and constitute an essential part of the innate immune system. Although the number of monocytes does not change significantly during ageing, several functional age-related changes in monocytes, such as altered expression of cytokines, defective Toll-like receptor signalling and a decreased capacity for phagocytosis, have been reported [6]. Monocytes are also involved in the initiation of atherosclerosis on arterial walls and have been linked to a chronic inflamed state (referred to as inflamm-ageing), which is associated with increased cardiovascular and metabolic diseases in elderly individuals [7]. Recent studies have revealed the important role of epigenetic regulation in the development and cell-specific functions of blood cells. Changes in DNA methylation patterns occur gradually throughout an individual’s lifespan [8,9] and may result in the age-related phenotypes of a specific set of genes [8]. The majority of these studies have examined DNA methylation changes in a mixed population of peripheral blood mononuclear cells (PBMCs) without purifying specific subsets of cells. In this study, we aimed to analyse the epigenomic changes in DNA methylation in purified monocyte cell populations from young and elderly individuals.

To study age-related DNA methylation profiles, we isolated monocytes from the peripheral blood of eight young (age range 22–25 years, mean 23.75 years; 4 females and 4 males) and eight elderly healthy volunteers (age range 77–78 years, mean 77.13 years; 4 females and 4 males). A whole genome methylation analysis was performed using the Infinium HumanMethylation450 BeadChip (Illumina Inc.). Altogether, we found 368 CpG sites that were significantly differentially methylated (p < 0.05), of which 26 CpG sites had an absolute β value differences greater than or equal to 0.2 between the young and old individuals (Table 1). Most of the CpG sites, a total of 21 positions, were hypomethylated in the elderly individuals; only five positions were hypermethylated in these individuals. Decreased methylation during the ageing process has been previously described in a study of PBMCs [10]. The most significantly altered sites mapped within the *NRP1, NRXN1, RASSF5, OTUD7A* and *PRM1* genes. The loci that did not reach the 0.2 β-difference threshold but were significantly different (p < 0.05) included two *ELOVL2* sites, cg16867657 and cg24724428 (both with β-diff. of 0.17); two *FHL2* sites, cg22454769 and cg24079702 (β-diff. of 0.15 and 0.14, respectively); and a *PENK* site, cg16419235 (β-diff. of 0.08); all these sites are associated with increased methylation in the peripheral blood mononuclear cells of older individuals [9,10].

**Table 1:** Differentially methylated sites in young versus old monocyte cell populations

**Target ID**	β-difference*	**Adjusted p-value**	**Gene**
**cg10501210**	**−0.38**	**0.002884**	***miR-29b-2*** * *
**cg24892069**	**−0.30**	**0.00312**	**NRP1**
**cg27209729**	**−0.30**	**0.020571**	**NRXN2**
cg11807280	−0.27	0.013523	
cg08128734	−0.27	0.015965	RASSF5
cg11693709	−0.26	0.024853	PAK6
cg18826637	−0.25	0.022927	
cg00329615	−0.25	0.012874	IGSF11
cg00740914	−0.25	0.008061	
cg03873281	−0.25	0.005647	PDLIM4
cg13039251	−0.23	0.008793	PDZD2
cg07583137	−0.23	0.007904	CHMP4C
cg12317815	−0.22	0.014773	ASPA
cg06781608	−0.22	0.030063	PTPRN2
cg13001142	−0.22	0.011913	STXBP5
cg16932827	−0.21	0.015062	
cg19344626	−0.21	0.010547	NWD1
cg14295611	−0.21	0.04857	
cg14614643	−0.21	0.008462	
cg03915012	−0.21	0.026415	GAK
cg03473532	−0.20	0.035003	MKLN1
cg02978201	0.47	0.015062	PRM1
cg04875128	0.30	0.017558	OTUD7A
cg21184711	0.23	0.024671	CADPS2
cg20665157	0.22	0.008629	CADPS2
cg19907915	0.21	0.014934	IGSF9B

The three replicated CpG loci are shown in bold.

*A negative β-difference indicates to hypomethylation in the elderly and a positive β-difference indicates to hypermethylation in the elderly population. Only CpG sites with FDR-adjusted p-values less than 0.05 were considered differentially methylated.

**Approximately 1 kb upstream.

To validate our results, we focused our investigation on the three differentially methylated CpG sites with the highest hypomethylation values, cg24892069, cg27209729 and cg10501210. The CpG site cg24892069, which had a very low standard deviation in both age groups (young STDEV: 0.05; old STDEV: 0.06), is located in intron 2 of the neuropilin 1 (*NRP1*) gene. NRP1 is a cell surface receptor with functional roles in several biological processes, including angiogenesis, immune response and regulation of vascular permeability [11,12], and has also been associated with increased cancer progression [13,14]. NRP1 is expressed in regulatory T cells [15] and is needed for prolonged cellular contact between regulatory T-cells and dendritic cells [16]. Another CpG site, cg27209729, is located in intron 9 of the neurexin 2 (*NRXN2*) gene. NRXN2 is a member of the neurexin family, which affects synaptic plasticity and cognitive functioning [17], and has been linked to autism spectrum disorders and schizophrenia [18]. The third CpG site, cg10501210, is located in putative regulatory region, approximately 1 kb upstream of the miR-29b-2 gene. miR-29b-2 belongs to the miR-29 family, which is important in thymic involution [19], T cell polarisation [20] and oncogenesis [19,21]. The miR-29b has been shown to target DNA methyltransferases DNMT3A and DNMT3B, and indirectly DNMT1 [22,23], leading to reduction of global methylation and expression of methylation regulated genes.

**Figure 1:**
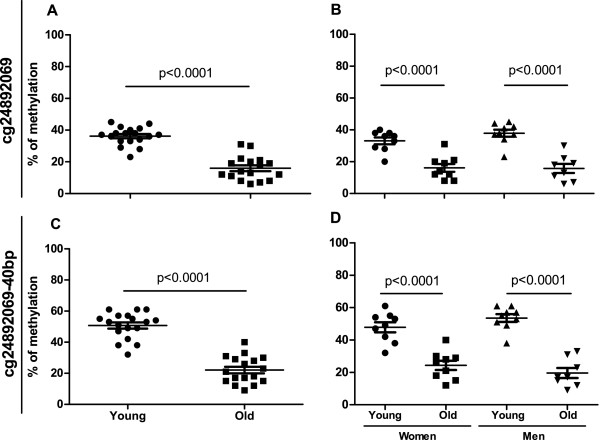
**Percentage of methylation of CpG sites cg24892069 (*NRP1; *A & B) and cg24892069-40 bp (*NRP1; *C & D).** The mean (±S.E.M.) methylation difference measured in 18 young and 17 old individuals using the Sequenom’s EPITYPER assay.

**Figure 2:**
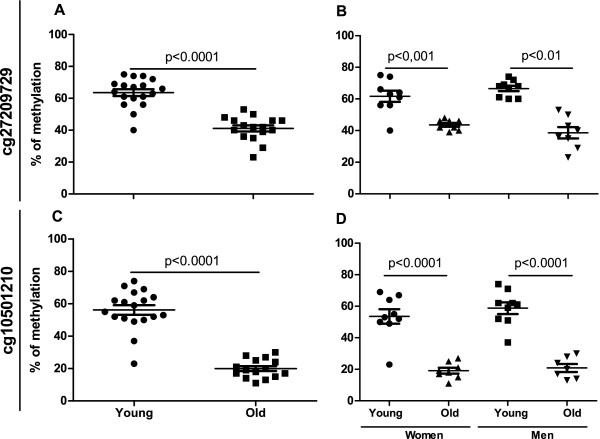
**Percentage of methylation of CpG sites cg27209729 (*NRXN2; *A & B) and cg10501210 (miR-29b-2; C & D).** The mean (±S.E.M.) methylation difference measured in 18 young and 15 old individuals using the Sequenom’s EPITYPER assay.

We replicated the array results of the three differentially methylated loci using the EpiTYPER assay (Sequenom Inc.) with a separate set of young and elderly samples. We added to our analyses two sex-matched control age groups, consisting of 10 young (age range 24–28 years, mean 26.4 years; 5 men and 5 females) and 10 elderly (age range 76–84 years, mean 79.4 years; 5 men and 5 females) samples. Using the EpiTYPER assay, we found hypomethylation of the NRP1-associated cg24892069 site in the monocytes of the older individuals, similar to the results from the HumanMethylation450 BeadChip analysis (Figure 1A). We also analysed the methylation differences in men and women separately and observed a significant difference in both gender groups (p < 0.0001) (Figure 1B). To explore this region further, we selected another CpG site, cg24892069-40 bp, which was located 40 bp upstream of the cg24892069 site in the genomic sequence; this site was not included on the methylation BeadChip. We found that the cg24892069-40 bp site had a statistically significant methylation difference between the studied age groups (p < 0.0001) (Figure 1C) and that was observed in both sexes (p < 0.0001) (Figure 1D). The similar DNA methylation pattern of the two CpG sites in close proximity is most likely the result of a shared, differentially methylated, region that is modified from the nearby methyltransferase binding site. We also found significant differences between the age groups at the cg27209729 and cg10501210 sites, located in the *NRXN2* gene and upstream of the miR-29b-2 gene, respectively (Figure 2). These CpG sites had statistically significant methylation differences in the combined study group (p < 0.0001) (Figure 2 A&C) as well as in the male and female study groups (p < 0.01 and p < 0.0001, respectively) (Figure 2 B&D).

We also evaluated the expression of the three differentially methylated CpG sites in monocytes of young and elderly individuals, but the expression levels *NRP1* and *NRXN2* genes were under the detection limit of RT-PCR. This is in agreement with our previously published mRNA expression study, where NRP1 was expressed at very low levels in monocytes and demonstrated a significantly increased expression in monocyte-derived dendritic cells and macrophages, whereas NRXN2 expression remained low even after the differentiation to dendritic cells [24]. The mRNA level of miR-29b-2 gene was detectable, however, the expression between young and elderly individuals did not differ significantly (data not shown). As the CpG site cg10501210 is located approximately 1 kb upstream of miR-29b-2 gene, it might not have regulatory effect on miR-29b-2 gene expression.

In conclusion, we were able to identify age-related DNA methylation changes in purified monocytes at immunologically relevant genomic loci. We found that the majority of the altered CpG sites were hypomethylated in the elderly individuals. The top three hypomethylated CpG sites in the elderly were cg24892069, cg27209729 and cg10501210, which are located in or near the *NRP1, NRXN2* and miR-29b-2 genes, respectively. Further investigation and a larger sample set are needed to define the functional role and significance of these CpG sites in the ageing process.

## Material and methods

### Purification of cell populations

The study is approved by Ethics Review Committee on Human Research of the University of Tartu. All of the participants gave written informed consent. Peripheral blood was obtained from healthy donors of Estonian Genome Center of University of Tartu. Peripheral blood mononuclear cells (PBMC) were extracted using a Ficoll-Paque (GE Healthcare) gradient centrifugation. CD14^+^ monocytes were extracted from PBMCs using microbeads (CD14+ #130-050-201) and AutoMACS technology (Miltenyi Biotec). The purity of monocyte cell population was analysed with FACSCalibur (BD Biosciences) using fluorescence conjugated antibodies against CD14 and CD3 (Miltenyi) to confirm the characteristic phenotype (Additional file 1: Figure S1).

### DNA extraction, bisulfite treatment and DNA methylation measurement

Genomic DNA was isolated from cell pellets using QIAmp DNA Micro Kit (Qiagen). DNA concentration was measured with NanoDrop ND-1000 spectrophotometry. Extracted genomic DNA was bisulfite converted using EZ-96 DNA Methylation Kit (Zymo Research Corporation). DNA methylation analysis was performed using Infinium Human Methylation 450 K bead chip technology (Illumina).

### Sequenom EpiTYPER assay

The Sequenom EpiTYPER technology was used to validate HumanMethylation450 array data. Samples were prepared using EpiTYPER T Complete Reagent Set (Sequenom) according to manufacturer’s instructions. 25 ng of bisulfite-treated DNA was used as PCR input and CpG methylation was determined by the MassARRAY Analyzer 4 system (Sequenom).

### Data analyses

The methylation signals were extracted with the methylation module v1.8.5 of the GenomeStudio v2010.3 software (Illumina Inc.) without background correction and normalisation. Probes with a detection p-value greater than 0.01, located on sex chromosomes or containing SNPs with a minor allele frequency of at least 5% in the Caucasian population according to the Hapmap project (http://hapmap.ncbi.nlm.nih.gov) were filtered out prior further analysis. The signals were corrected and normalised using subset quantile normalisation as described in [25]. For differential methylation analysis, 80% of the least varying probes according to interquartile range across all samples were removed and a linear model was used to assess the differences between two age groups considering arrays on different BeadChips as batches. Methylation sites with a FDR adjusted p-value less than 0.05 were considered differentially methylated. Median difference of beta values greater than 0.2 between groups was considered for selecting methylation sites for further analyses.

## Competing interests

The authors declare that they have no competing interests.

## Authors’ contributions

TL and PP designed the study and wrote the manuscript; TL purified the cells and carried out the methylation array; LM carried out the validation experiments; SM analysed the data and helped to interpret the data; KK, MA and ML contributed to the design of the study and coordinated the recruitment of the study participants. All authors read and approved the final manuscript.

## Supplementary Material

Additional file 1: Figure S1 The purification of monocyte cell population. The monocytes were analysed with FACSCalibur (BD Biosciences) using fluorescence conjugated antibodies against CD14 and CD3 (Miltenyi). Click here for file
