# Supplementary material for: CpG sites associated with NRP1, NRXN2 and miR-29b-2 are hypomethylated in monocytes during ageing
Source: Immun Ageing. 2014 Jan 9;11:1. doi: 10.1186/1742-4933-11-1 (PMC3896835; doi:10.1186/1742-4933-11-1)
Supplement: Additional file 1: Figure S1 — The purification of monocyte cell population. The monocytes were analysed with FACSCalibur (BD Biosciences) using fluorescence conjugated antibodies against CD14 and CD3 (Miltenyi). [file 1742-4933-11-1-S1.pdf]

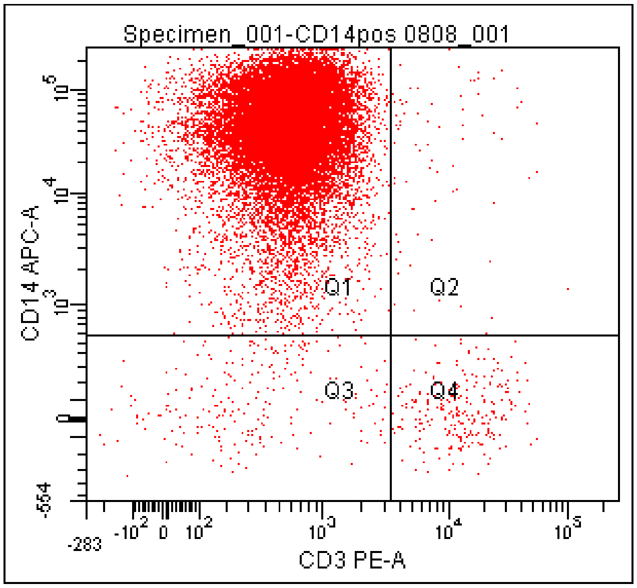


**Additional file 1: Figure S1. The purification of monocyte cell population.** The monocytes were analysed with FACSCalibur (BD Biosciences) using fluorescence conjugated antibodies against CD14 and CD3 (Miltenyi).
